# A state-dependent dilatancy constitutive model and parametric analysis for dense sand–structure interfaces

**DOI:** 10.1371/journal.pone.0358465

**Published:** 2026-09-16

**Authors:** Xiaolin Li, Chunlin Wang, Jugang Luo, Gaofeng Hou, Daguo Wu, Ge Zhang

**Affiliations:** 1 Anhui and Huaihe River Institute of Hydraulic Research, Hefei, China; 2 Anhui Construction Engineering Quality Supervision and Inspection Station Co., Ltd, Hefei, China; 3 Anhui Provincial Key Laboratory of Water Science and Intelligent Water Conservancy, Water, Hefei, China; 4 Key Laboratory of Geological Hazards on Three Gorges Reservoir Area, Ministry of Education, China Three Gorges University, Yichang, Hubei, China; 5 Hubei Engineering Technology Research Center for Disaster Prevention and Mitigation, China Three Gorges University, Yichang, Hubei, China; 6 Hubei Provincial Engineering Research Center of Slope Habitat Construction Technique Using Cement-based Materials, China Three Gorges University, Yichang, Hubei, China; University of Sharjah, UNITED ARAB EMIRATES

## Abstract

To describe the pronounced dilatancy, normal-stress evolution under constrained conditions, and post-peak softening of dense sand–structure interfaces, this paper develops a simplified state-dependent dilatancy-coupled constitutive model within the framework of critical-state soil mechanics. The state parameter is introduced to characterize the density state of the interface, while the dilatancy rate is used to describe the normal plastic deformation induced by interface shearing. Under constant normal stiffness (CNS) conditions, the constrained dilatancy is converted into an increment of normal stress, enabling the model to continuously capture the complete interface response from pre-peak hardening and peak mobilization to post-peak softening. An incremental numerical scheme is formulated, and the model is calibrated and evaluated through comparisons with published CNS direct-shear test results for dense sand–structure interfaces. The comparisons show that the proposed model can reasonably reproduce the main characteristics of interface shearing, including the evolution of shear stress, the increase in normal stress, and post-peak strength degradation. Parametric analyses indicate that the critical-state-line parameters and the initial void ratio primarily govern the initial dilatancy potential of the interface; the dilatancy coefficient has the most significant influence on the peak shear strength and the magnitude of normal-stress increase; and the shear-band thickness mainly controls the rate of state evolution and the post-peak response. The proposed model provides a concise constitutive framework for describing the coupled evolution of dilatancy, normal stress, and shear strength of dense sand–structure interfaces under CNS conditions.

## 1. Introduction

The mechanical behavior of sand–structure interfaces plays an important role in a wide range of geotechnical systems, including pile foundations, retaining structures, underground structures, and other soil–structure interaction problems. For dense sand–structure interfaces, particle interlocking, rolling, and rearrangement during shearing generally produce pronounced dilatancy. The resulting interface response is strongly affected by the imposed normal boundary condition. Under constant normal load (CNL) conditions, normal stress remains approximately unchanged while the interface is allowed to dilate. Under constant normal stiffness (CNS) conditions, however, normal expansion is restrained by the surrounding medium and is converted into an increase in normal stress. This increase in normal stress enhances interface shear resistance, whereas the progressive attenuation of dilatancy with continued shearing may lead to the development of peak strength followed by post-peak softening. Therefore, an appropriate constitutive description of dense sand–structure interfaces should account for the coupled evolution of density state, dilatancy, normal stress, and shear strength.

Experimental investigations have provided important insights into the factors governing soil–structure interface behavior. Early studies commonly employed CNL direct-shear or simple-shear tests to examine the fundamental frictional response of sand–structure interfaces. Jardine et al. systematically investigated friction coefficients between different sands or silts and steel surfaces representative of industrial piles [1]. Based on instrumented pile tests, Lehane et al. demonstrated that interface shear resistance in sand is closely related to dilatancy, changes in radial effective stress, and restructuring of the surrounding soil fabric [2]. Evgin and Fakharian examined the influence of stress path on sand–steel interface response through simple-shear tests [3]. Porcino et al. conducted direct-shear tests on sand–structure interfaces under different normal boundary conditions and showed that interface roughness, normal stress level, and relative density are major factors controlling the frictional response [4]. Using particle image velocimetry, DeJong et al. found that interface shear deformation is generally concentrated within a thin shear band with a thickness of approximately five to seven times the mean particle diameter [5].

Further experimental studies have examined the effects of surface properties, construction procedures, temperature, and loading conditions. Zhao et al. established a relationship between surface hardness and the peak interface friction coefficient under different particle-size conditions [6]. Shi et al. and Wan et al. investigated the influence of post-grouting parameters on interface mechanical behavior and showed that the degree of normal constraint can significantly affect the strengthening response [7,8]. Di Donna et al. examined soil–concrete interfaces under cyclic and thermal loading and reported distinct responses for sand–concrete and clay–concrete interfaces [9]. Wang et al. studied the effects of temperature variation and thermal cycling on the frictional behavior of kaolin–concrete interfaces [10]. Wang et al. and Lin et al. investigated interface behavior under warm-permafrost and seasonally frozen conditions, respectively, and demonstrated that temperature can have a significant influence on interface strength and deformation characteristics [11,12]. These studies indicate that interface behavior is controlled by multiple factors, among which the normal boundary condition is particularly important for dense granular materials. Interface tests under CNS conditions have consequently received increasing attention. Fakharian and Evgin [13] revealed that CNS constraints substantially alter the interface stress path and volumetric response compared with conventional CNL conditions. Lashkari showed that constrained dilatancy under CNS conditions can cause a continuous increase in normal stress and thereby modify the mobilization and evolution of interface shear strength [14]. Zhou et al. and Zhang et al., through combined experimental and discrete-element investigations, further examined the coupled macro- and meso-scale mechanisms of interface behavior under cyclic loading and dynamic normal loading [15,16]. These findings demonstrate that the interaction between interface dilatancy and normal constraint is essential for describing the pre-peak strengthening, peak formation, and post-peak response of dense sand–structure interfaces.

In terms of constitutive modeling, interface models have gradually evolved from empirical stress–displacement relationships to formulations incorporating plasticity, critical-state concepts, and state dependence. Early load-transfer approaches, such as the 𝑡-𝑧 method, commonly represented the relationship between interface shear stress and relative displacement using hyperbolic or piecewise linear curves [17]. Although such approaches are convenient for engineering calculations, they are essentially empirical and generally cannot represent differences in dilatancy caused by variations in the initial density state. Boulon and Foray introduced CNS conditions into interface studies and demonstrated that interface dilatancy can significantly affect the mobilized shear resistance [18]. Ghionna and Mortara developed an elastoplastic model for sand–structure interfaces that considered the interaction between interface deformation and normal stress [19]. The development of state-dependent sand models provides a useful theoretical basis for describing density-dependent interface behavior. Li and Wang proposed a critical-state-line relationship and introduced the state parameter, defined as the difference between the current void ratio and the critical-state void ratio under the same stress condition, namely $\Psi =e-e_{c}$ [20]. Li and Dafalias subsequently expressed dilatancy as a state-dependent variable within a critical-state framework, enabling the responses of loose and dense sands to be represented using a unified formulation [21]. Lashkari introduced state-dependent concepts into sand–structure interface modeling and proposed a constitutive model applicable to interfaces with different initial densities under both CNL and CNS conditions [14]. Lashkari proposed a simple critical-state interface model and applied it to predict the shaft resistance of non-displacement piles in sand [22]. Liu et al. proposed a three-dimensional state-dependent generalized-plasticity model for soil–structure interfaces under monotonic and cyclic loading [23]. Zhou et al. combined an interface constitutive model with a load-transfer approach to investigate the load–settlement response of piles in sand [24]. Peng et al. systematically investigated the dilatant behavior of sand–pile interfaces during loading and stress relief and demonstrated the influence of stress history on the interface volumetric response [25]. Existing empirical models cannot adequately capture the effects of initial density on interface dilatancy and post-peak softening. Meanwhile, rigorous state-dependent elastoplastic models often involve numerous parameters and complex calibration procedures.

Numerical and analytical methods have also been widely used to study soil–structure interaction and interface behavior. In continuum analyses, the interface is commonly represented using zero-thickness contact elements or thin-layer elements, together with Coulomb-type friction laws or specialized interface constitutive relationships. Rui and Yin employed a coupled thermo-hydro-mechanical finite-element method to investigate pile–soil interaction under thermal and mechanical loading and showed that cyclic thermal deformation can significantly affect interface shear mobilization [26]. Wu et al. proposed a simplified method for determining interface parameters for vertically loaded piles in layered soils using FLAC3D [27]. Mascarucci et al. developed a numerical approach that explicitly considered shear-band behavior, interface dilatancy, and critical-state response in the estimation of shaft friction for bored piles in sand [28]. Analytical and numerical approaches have also been developed for pile–soil interaction problems involving negative skin friction, dynamic loading, frozen-soil foundations, and soil-arching effects [29–32]. Discrete-element methods provide an alternative means of investigating the particle-scale mechanisms associated with interface shearing and granular rearrangement. Lobo-Guerrero and Vallejo used discrete-element simulations to study particle crushing around driven piles and demonstrated the influence of pile geometry on deformation and crushing patterns in granular materials [33]. Butlanska et al. developed a three-dimensional virtual calibration chamber to simulate soil deformation and stress redistribution during cone penetration [34]. Arroyo et al. subsequently conducted a multiscale investigation of cone penetration in virtual calibration chambers and examined the effects of model size and boundary conditions on penetration resistance [35]. In addition, numerical platforms such as 3DEC provide user-defined scripting functions that allow contact properties and constitutive parameters to be updated during the calculation cycle [36]. These developments demonstrate the importance of interface constitutive models that are physically interpretable, computationally concise, and suitable for incremental numerical integration. Nevertheless, before such models are extended to complex engineering-scale simulations, their ability to reproduce the fundamental stress–displacement response of sand–structure interfaces should first be established and verified at the interface level.

Despite substantial progress, existing empirical models cannot adequately describe the coupled effects of density-dependent dilatancy, normal-stress evolution, and post-peak softening at sand–structure interfaces. More rigorous state-dependent models, however, often involve numerous parameters and complex calibration or integration procedures. Accordingly, this paper develops a simplified state-dependent dilatancy-coupled constitutive model for dense sand–structure interfaces under CNS conditions. The state parameter Ψ is adopted as the core variable characterizing the density state of the interface, and a state-dependent dilatancy relationship is introduced to determine the normal plastic deformation induced by interface shearing. The CNS boundary condition is used to convert constrained dilatancy into an increment of normal stress, thereby coupling the evolution of normal stress with the mobilized interface shear strength. An incremental stress-update procedure is formulated for the proposed constitutive equations. The model is then calibrated and validated against published CNS direct-shear test results for dense sand–structure interfaces. Finally, parametric analyses are conducted to investigate the effects of the critical-state-line parameters, dilatancy coefficient, shear-band thickness, and initial void ratio on the shear-stress and normal-stress responses of the interface. Compared with existing state-dependent interface models, the proposed model provides a simplified constitutive framework while retaining the key mechanisms governing dense sand–structure interface behavior. The model adopts a reduced parameter framework and explicitly couples state-dependent dilatancy with CNS-constrained normal stress evolution through an incremental stress-update scheme. In addition, an interface-opening criterion is incorporated to describe the transition from compressed contact to separation. This formulation enables the model to capture strength mobilization and post-peak softening behavior under CNS conditions while reducing calibration complexity.

## 2. Theoretical formulation of a state-dependent dilatancy-coupled model for dense sand–structure interfaces

Before establishing the constitutive relationship for dense sand–structure interfaces, it is necessary to clarify the fundamental volumetric response of dense sand during interface shearing. At the initial stage of shearing, strong particle interlocking causes relative particle movement to be accompanied by lifting, rolling, and rearrangement, resulting in pronounced dilatancy. As shear displacement accumulates, the initial dense fabric is progressively disturbed and the interface material gradually approaches the critical state. Consequently, the additional particle lifting required for continued shearing decreases, and the dilatancy capacity progressively attenuates. Once the critical state is reached, the particle arrangement becomes statistically stable, volumetric deformation tends to cease, and the interface enters a constant-volume shearing stage.

### 2.1. Constrained dilatancy and normal-stress evolution of dense sand–structure interfaces under CNS conditions

**Fig 1:**
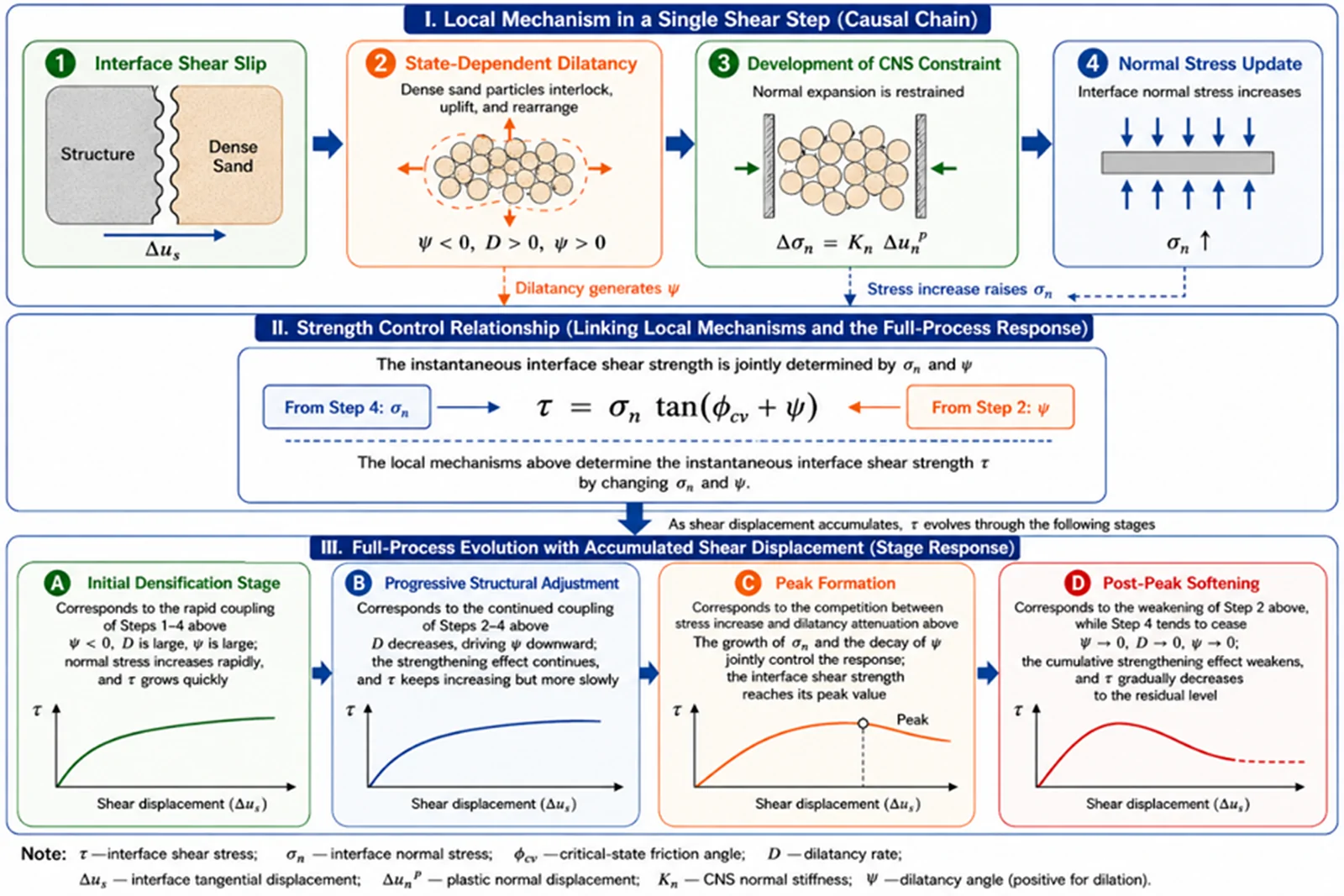
Schematic diagram of constrained dilatancy and strength evolution of dense sand–structure interfaces under CNS conditions.

The mechanical response of dense sand–structure interfaces is strongly influenced by the constant normal stiffness (CNS) condition, whose essential mechanism is the coupling between interface dilatancy and normal constraint. During shearing, particles near the interface undergo rolling and rearrangement, producing a tendency for normal dilation. Under CNS conditions, this dilation is restrained by the imposed normal stiffness($K_{n}$), and the corresponding normal displacement is converted into an increase in normal stress. Therefore, interface shearing involves a continuous coupled process of shear-induced dilatancy, constrained normal-stress increase, and enhancement of shear resistance. The peak interface strength is jointly governed by the initial density state, dilatancy capacity, and normal constraint stiffness. The coupled mechanism and the associated strength-evolution process are schematically illustrated in Fig 1.

To quantitatively describe the above coupling mechanism, the state-parameter concept from critical-state soil mechanics is introduced [20]. The critical state refers to a stable condition reached during continued shearing, at which volumetric deformation tends to cease while the shear stress remains approximately constant. The state parameter is defined as the difference between the current void ratio e and the critical-state void ratio $e_{c}$ under the same normal stress, namely $\Psi =e-e_{c}$. When Ψ<0, the interface material lies on the dense side of the critical state and tends to dilate during shearing; when Ψ=0, the critical state is reached and dilatancy tends to vanish. Li and Dafalias [21] formulated dilatancy as a function of the state parameter, enabling sands with different initial density states to be described within a unified constitutive framework.

In this study, the state-dependent dilatancy framework is applied to dense sand–structure interfaces, with the state parameter Ψ adopted as the principal variable governing the evolution of interface dilatancy. At the initial stage of shearing, strong particle interlocking and rearrangement cause the dense interface material to exhibit a pronounced tendency to dilate. Under CNS conditions, this normal expansion tendency is restrained by the imposed normal stiffness and converted into an increase in normal stress, thereby enhancing the interface shear strength. Therefore, the pre-peak strengthening of dense sand–structure interfaces results from the coupled effects of state-dependent dilatancy, constrained normal-stress increase, and frictional-strength enhancement. As shear displacement accumulates, the particle fabric within the interface shear band progressively evolves from the initial dense state toward the critical state, and the dilatancy capacity gradually decreases. This occurs because the initial dense particle fabric is continuously rearranged during shearing, reducing the additional particle lifting required for further deformation. Accordingly, the dilatancy rate is defined as:


$$\begin{array}{c} D=\frac{\text{d}u_{n}^{p}}{\text{d}u_{s}^{p}} \end{array}$$ (1)

where $u_{n}^{p}$ and $u_{s}^{p}$ denote the normal and tangential plastic displacements of the interface, respectively. As the critical state is approached, D gradually decreases toward zero [21]. Since the dynamic dilatancy angle satisfies:


$$\begin{array}{c} \text{tan}\psi =D \end{array}$$ (2)

The dilatancy angle ψ decreases accordingly and eventually tends to zero. The interface shear strength is jointly governed by the normal stress $\sigma_{n}$ and the mobilized friction angle. In the proposed model, the mobilized friction angle is related to the critical-state friction angle and the dilatancy angle. At the initial stage of shearing, the relatively large dilatancy rate produces a rapid increase in normal stress under the CNS constraint. Meanwhile, the dilatancy angle increases the mobilized friction angle, causing the interface shear stress to rise rapidly. As shearing continues, both the dilatancy rate and the dilatancy angle gradually decrease. When the reduction in dilatancy-induced strengthening exceeds the strength gain associated with the increase in normal stress, the interface shear strength reaches a peak and subsequently exhibits post-peak softening. As the interface approaches the critical state, namely Ψ→0, D→0, and ψ→0, dilatancy ceases and the interface shear strength gradually approaches a stable level governed jointly by the final normal stress and the critical-state friction angle $\varphi_{cv}$.

In summary, the proposed model uses the state parameter Ψ to characterize the density state of the interface and the dilatancy rate D to describe the normal plastic deformation induced by interface shearing. The CNS boundary condition converts the constrained normal plastic deformation into an increment of normal stress. In this manner, the model couples state evolution, dilatancy attenuation, normal-stress development, and shear-strength variation, thereby providing the physical basis for the state-evolution equation, dilatancy equation, CNS normal-stress update equation, and strength equation presented in the following sections.

### 2.2. Establishment of the interface constitutive equations

Based on the above physical mechanism, this section establishes an incremental constitutive equation system for dense sand–structure interfaces. The model adopts an elastoplastic framework, uses the Mohr–Coulomb criterion to describe yielding under compression-shear conditions, introduces state-dependent dilatancy theory to characterize normal plastic deformation induced by interface shearing, and updates the normal stress through the CNS boundary condition. In addition, to account for possible interface opening and separation, a normal tensile strength is introduced to truncate stress transfer after the interface opens.

#### 2.2.1. Elastic constitutive relationship.

The normal and tangential elastic responses of the interface are controlled by the normal stiffness $K_{n}$ and the tangential stiffness $K_{s}$, respectively. For given increments of normal displacement $\Delta u_{n}$ and tangential displacement $\Delta u_{s}$, the elastic stress increments can be written as


$$\begin{array}{c} \Delta \sigma_{n}^{e}=K_{n}\cdot \Delta u_{n},\;\Delta \tau^{e}=K_{s}\cdot \Delta u_{s} \end{array}$$ (3)

In the purely elastic stage, the interface normal stress and shear stress are incrementally accumulated and updated according to Eq. (3).

#### 2.2.2. Yield criterion and plastic sliding condition.

The yielding behavior of the interface under compression-shear conditions is described by the Mohr–Coulomb criterion, and the yield function is defined as


$$\begin{array}{c} f=|\tau |-\sigma_{n}\cdot \text{tan}\phi_{m} \end{array}$$ (4)

where $\phi_{m}$ is the mobilized friction angle of the interface, expressed as,


$$\begin{array}{c} \phi_{m}=\phi_{cv}+\psi \end{array}$$ (5)

where $\phi_{cv}$ is the critical-state constant-volume friction angle and ψ is the dynamic dilatancy angle.

When the interface is in a compressed contact state and f≤0, the interface remains elastic. When f>0, plastic sliding occurs, the plastic tangential displacement increment is,


$$\begin{array}{c} \Delta u_{s}^{p}=\frac{\left|\tau_{tr}\right|-\sigma_{n}\text{tan}\phi_{m}}{K_{s}} \end{array}$$ (6)

where $\tau_{tr}=\tau_{old}+K_{s}\Delta u_{s}$ is the elastic trial shear stress. After return mapping, the shear stress is taken as,


$$\begin{array}{c} \tau =\text{sign}\left(\tau_{tr}\right)\cdot \sigma_{n}\text{tan}\phi_{m} \end{array}$$ (7)

#### 2.2.3. State-dependent dilatancy equation.

After plastic sliding occurs, the normal plastic deformation of the interface is controlled by the dilatancy rate D. According to the state-dependent dilatancy theory proposed by Li and Dafalias [21], the dilatancy rate can be expressed as a function of the state parameter Ψ:


$$\begin{array}{c} D=\frac{du_{n}^{p}}{du_{s}^{p}}=-A\cdot \Psi \end{array}$$ (8)

where A is the dilatancy coefficient. The state parameter satisfies $\Psi =e-e_{c}$, where the critical-state void ratio $e_{c}$ is determined by the relationship proposed by Li and Wang [20]:


$$\begin{array}{c} e_{c}=e_{\Gamma }-\lambda {\left(\frac{\sigma_{n}}{P_{a}}\right)}^{\xi } \end{array}$$ (9)

where $e_{\Gamma }$, λ, and ξ are critical-state-line material constants, and $P_{a}$ is the reference atmospheric pressure.

When Ψ<0 (dense side), D>0 and the interface dilates. When Ψ→0 (approaching the critical state), D→0 and dilatancy tends to cease.

The dynamic dilatancy angle can further be obtained from the dilatancy rate as


$$\begin{array}{c} \psi =\text{arctan}(D) \end{array}$$ (10)

The corresponding normal plastic displacement increment is,


$$\begin{array}{c} \Delta u_{n}^{p}=D\cdot \Delta u_{s}^{p} \end{array}$$ (11)

#### 2.2.4. Normal-stress updating under the CNS constraint.

Under CNS boundary conditions, the normal plastic expansion of the interface is constrained by the surrounding soil and converted into additional normal stress:


$$\begin{array}{c} \sigma_{n(new)}=\sigma_{n(old)}+K_{n}\cdot \Delta u_{n}^{p} \end{array}$$ (12)

In this study, compressive normal stress is defined as positive, positive normal displacement indicates interface dilation, and D>0 denotes dilatancy.

Equ. (12) reflects the “dilatancy–normal stress increase” coupling mechanism: normal expansion generated by plastic shearing is converted into a normal stress increment under the CNS constraint, which further increases the shear resistance of the interface through the yield-strength expression.

#### 2.2.5. Normal tensile strength and interface opening condition.

In practice, the pile–soil interface can transmit only a limited tensile normal action. When the interface normal stress gradually changes from compression to tension and the tensile normal stress reaches the specified normal tensile strength $\sigma_{t}$, the interface opens and separates, and the contact relationship fails. Therefore, the following tensile truncation condition is introduced:


$$\begin{array}{c} \sigma_{n}\leq \sigma_{t} \end{array}$$ (13)

where $\sigma_{t}$ is the upper limit of the interface normal tensile strength. Considering that once the interface opens it loses the compaction and interlocking effects and can no longer transmit tangential friction, when the computed normal stress exceeds the specified tensile limit, the interface stress is treated according to the opening condition as


$$\begin{array}{c} \sigma_{n}=\text{0},\;\;\tau =\text{0} \end{array}$$ (14)

The above treatment indicates that after the contact surface enters the open state, the interface no longer transmits normal pressure or tangential shear stress. Only when subsequent displacement evolution causes the interface to close again and recover a compressed contact state are interface friction and dilatancy coupling reactivated. Therefore, the proposed model can describe not only dilatancy-induced normal stress increase and strength enhancement under compression, but also the interface opening and separation condition. It should be noted that the interface-opening criterion introduced in this study is mainly used as a numerical contact-state criterion to describe the transition from compression contact to separation when the tensile limit is reached. The present validation focuses on the coupled evolution of dilatancy, normal stress, and shear strength under CNS compression-shear conditions, while the detailed investigation of interface opening behavior under tensile or cyclic loading conditions requires further validation.

#### 2.2.6. Evolution of interface void ratio.

The void ratio within the interface shear band is dynamically updated with normal plastic deformation. Introducing the shear-band thickness t (usually taken as 5–10 times the mean particle diameter $d_{\text{5}\text{0}}$), the void-ratio increment can be written as,


$$\begin{array}{c} e_{new}=e_{old}+\frac{\left(\text{1}+e_{\text{0}}\right)}{t}\cdot \Delta u_{n}^{p} \end{array}$$ (15)

The change in void ratio affects $e_{c}$ through Eq. (9), thereby updating the state parameter Ψ and causing the dilatancy rate D to gradually attenuate with accumulated shear, ultimately driving the interface to transition from peak strength to residual strength.

#### 2.2.7. Coupling relationship and closure of the model equations.

**Fig 2:**
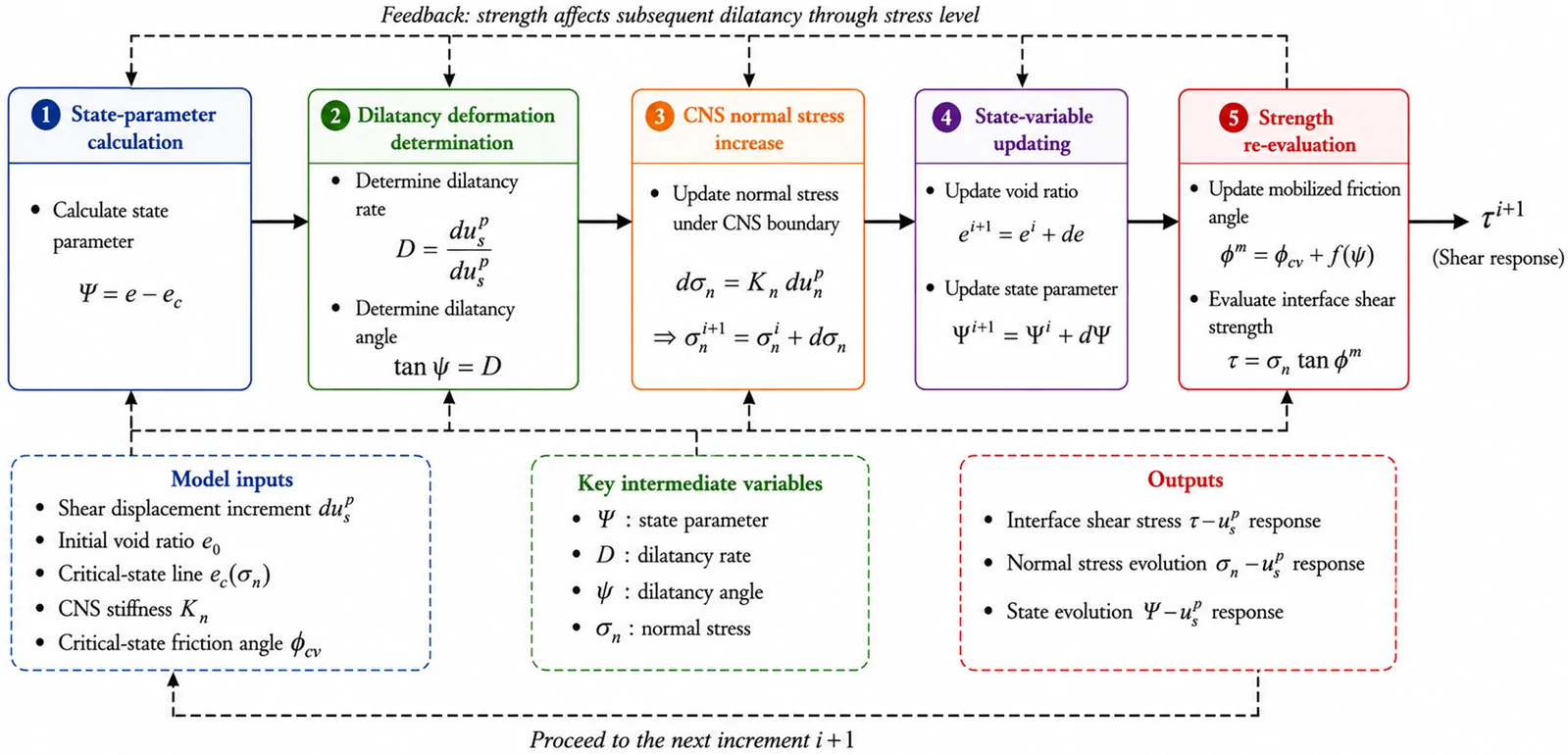
Coupling relationship and closure mechanism of the proposed constitutive model.

The above equations are mutually coupled and together form a closed constitutive system. The closure of the model is achieved through the feedback among the density state, dilatancy response, normal-stress evolution, and shear-strength mobilization. As shown in Fig 2, the current normal stress $\sigma_{n}$ and void ratio e are first used to determine the critical-state void ratio $e_{c}$ and the state parameter Ψ. The state parameter Ψ then controls the dilatancy rate D and the dynamic dilatancy angle ψ. During plastic sliding, the dilatancy rate D determines the normal plastic displacement increment $\Delta u_{n}^{p}$, which is converted into a normal-stress increment through the CNS boundary condition. The updated normal stress $\sigma_{n}$ and void ratio e further modify the state parameter Ψ and the subsequent dilatancy response. Meanwhile, the normal stress $\sigma_{n}$ and dilatancy angle ψ jointly determine the mobilized interface shear strength. Therefore, the core closure process of the model can be summarized as: state-parameter calculation, dilatancy determination, CNS normal-stress updating, state-variable evolution, and shear-strength re-evaluation.

Specifically, the current normal stress $\sigma_{n}$ and void ratio e jointly determine the critical-state void ratio $e_{c}$ and the state parameter Ψ. The state parameter Ψ further controls the dilatancy rate D and the dynamic dilatancy angle ψ. During plastic sliding, D determines the normal plastic displacement increment, whereas ψ contributes to the mobilized friction angle and the corresponding yield strength. Therefore, the state parameter affects not only the normal deformation tendency of the interface, but also the mobilized shear strength through the dilatancy angle.

Under CNS conditions, the normal plastic deformation induced by dilatancy is restrained by the imposed normal stiffness and converted into an increment of normal stress. The updated normal stress $\sigma_{n}$ directly affects the interface shear strength and also changes the critical-state void ratio $e_{c}$ through the critical-state line in the next calculation step. Meanwhile, the normal plastic deformation updates the void ratio e within the interface shear band, causing the state parameter Ψ to evolve continuously during shearing. Consequently, $\sigma_{n}$, e, Ψ, D, and ψ are mutually updated in each incremental step and serve as the initial state variables for the subsequent step, forming a closed feedback loop among state evolution, dilatancy deformation, normal-stress development, and strength mobilization.

The interface yield strength is jointly governed by the normal stress and the dilatancy angle and can be expressed as


$$\begin{array}{c} \tau_{y}=\sigma_{n}\text{tan}(\varphi_{cv}+\psi ) \end{array}$$ (16)

where $\sigma_{n}$ represents the normal stress acting on the interface, $\varphi_{cv}$ is the critical-state friction angle, and ψ reflects the contribution of state-dependent dilatancy to the mobilized friction angle. These variables jointly determine the yield strength in the current step and feed back into the subsequent yield judgment and stress-update process. When the interface enters the tensile region and the tensile limit $\sigma_{t}$ is exceeded, both the normal stress and tangential shear stress are truncated to zero. Once the interface closes again, frictional sliding, dilatancy deformation, and the CNS normal-stress updating mechanism are reactivated.

Thus, the proposed model achieves closure of the constitutive equation system through the mutual feedback among $\sigma_{n}$, e, Ψ, D, ψ, and $\tau_{y}$. The model can continuously describe the response of dense sand–structure interfaces under CNS conditions, including dilatancy-induced normal-stress increase, peak-strength formation, post-peak softening, and the boundary case of interface opening and separation. The model involves nine parameters: the critical-state-line parameters $e_{\Gamma }$, λ, and ξ; the friction parameter $\varphi_{cv}$; the dilatancy coefficient A; the stiffness parameters $K_{n}$ and $K_{s}$; the shear-band thickness t; and the normal tensile strength $\sigma_{t}$. These parameters have clear physical meanings and can be calibrated through conventional geotechnical tests or interface shear tests.

### 2.3. Numerical integration procedure of the constitutive model

To facilitate the calculation of the proposed constitutive model, an incremental stress-update procedure is formulated. In each loading increment, the state variables, dilatancy variables, and stress variables are updated sequentially according to the coupled constitutive equations. The calculation procedure is as follows:

1 **State variable acquisition:** At the beginning of the current increment, the normal stress $\sigma_{n}$, shear stress τ, void ratio e, and tangential displacement increment $\Delta u_{s}$ of the interface are obtained.
2 **Critical-state and state-parameter calculation:** The current critical-state void ratio $e_{c}$ is calculated from the critical-state relationship using the normal stress $\sigma_{n}$. The state parameter is then obtained as $\Psi =e-e_{c}$.
3 **Dilatancy and strength calculation:** The dilatancy rate D is calculated from the state parameter Ψ, and the dynamic dilatancy angle ψ is determined from tanψ=D. The current interface yield strength is then calculated as:

$$\begin{array}{c} \tau_{f}=\sigma_{n}\text{tan}(\varphi_{cv}+\psi ) \end{array}$$ (17)

4 **Elastic trial and yield judgment:** The trial shear stress is calculated as

$$\begin{array}{c} \tau_{tr}=\tau_{old}+K_{s}\Delta u_{s} \end{array}$$ (18)

If $\text{abs}(\tau_{tr})\leq \tau_{f}$, the interface remains in the elastic state, and $\tau_{new}=\tau_{tr}$, $\Delta u_{s}^{p}=\text{0}$. If $\text{abs}(\tau_{tr})>\tau_{f}$, plastic correction is performed.

5 **Plastic correction:** For the yielded state, the plastic tangential displacement increment is calculated as

$$\begin{array}{c} \Delta u_{s}^{p}=\frac{\text{max}(\text{abs}(\tau_{tr})-\tau_{f},\text{0})}{K_{s}} \end{array}$$ (19)

and the shear stress is returned to the yield surface.

6 **Normal plastic deformation calculation:** The normal plastic displacement increment induced by dilatancy is calculated as

$$\begin{array}{c} \Delta u_{n}^{p}=D\Delta u_{s}^{p} \end{array}$$ (20)

7 **Normal-stress and state-variable update:** Under the CNS condition, the normal plastic displacement increment is converted into a normal-stress increment:

$$\begin{array}{c} \sigma_{n}^{new}=\sigma_{n}^{old}+K_{n}\Delta u_{n}^{p} \end{array}$$ (21)

Meanwhile, the void ratio within the interface shear band is updated according to the normal plastic deformation and the shear-band thickness t. If the updated normal stress enters the tensile region and exceeds the tensile limit $\sigma_{t}$, the interface is regarded as open, and both the normal stress and shear stress are set to zero, namely $\sigma_{n}=\text{0}$ and τ=0.

8 Parameter update for the next increment: Based on the updated $\sigma_{n}$ and e, the critical-state void ratio $e_{c}$, state parameter Ψ, dilatancy rate D, dilatancy angle ψ, and yield strength are recalculated. These updated variables are then used as the initial state for the next loading increment.

The above procedure forms a closed incremental loop of elastic trial, yield judgment, plastic correction, dilatancy-induced normal-stress updating, state-variable evolution, and strength re-evaluation. This stress-update procedure provides a convenient numerical integration scheme for the proposed state-dependent dilatancy-coupled model and can be used to simulate the stress–displacement response of dense sand–structure interfaces under CNS conditions.

## 3. Model verification and parametric analysis of dense sand–structure interfaces

### 3.1. Model verification based on CNS direct-shear tests of sand–structure interfaces

**Fig 3:**
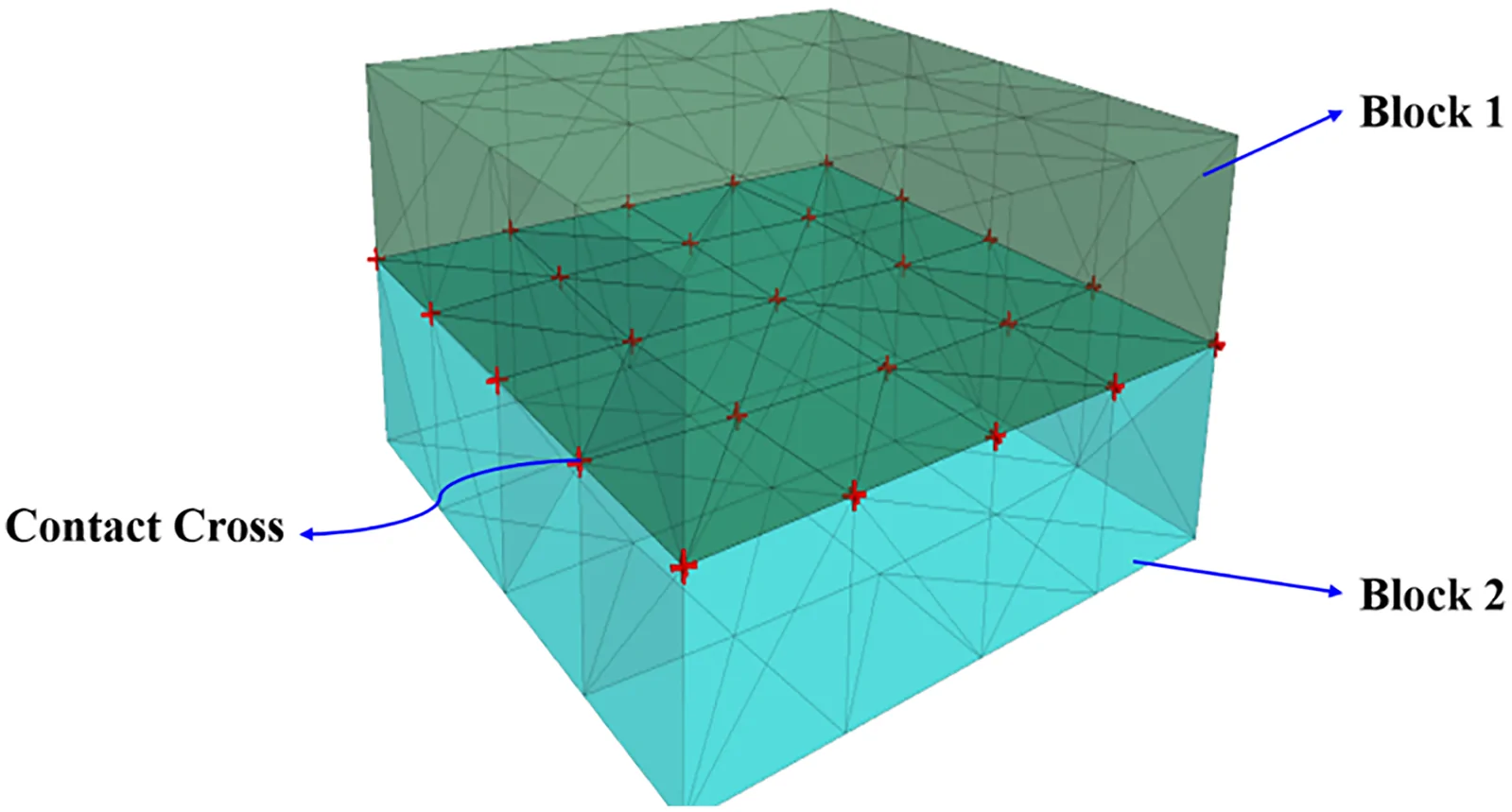
Schematic diagram of the computational model.

To evaluate the ability of the proposed state-dependent dilatancy-coupled model to describe the shearing behavior of dense sand–structure interfaces under constant normal stiffness (CNS) conditions, the CNS direct-shear test results reported by Porcino et al. [4] are used for model calibration and comparison. In that study, a CNS direct-shear apparatus was employed to investigate the frictional behavior of three natural silica sands against aluminum plates with four different roughness levels, and the results were compared with those obtained under constant normal load (CNL) conditions. The reported results indicate that the interface shear response is mainly governed by normal constraint stiffness, interface roughness, sand properties, relative density, and initial effective normal stress [4]. Considering the focus of the present study on dense sand–structure interfaces, representative CNS test conditions are selected to examine whether the proposed model can capture the typical features associated with dense-sand interface shearing, including constrained dilatancy, normal-stress increase, peak-strength mobilization, and post-peak softening. It should be noted that the purpose of this section is not to achieve a complete one-to-one fitting for a specific material combination, but to evaluate the capability of the proposed model to reproduce the fundamental coupling mechanisms observed in measurable CNS direct-shear tests. Therefore, the verification focuses on the general evolution of interface state, normal stress, and shear strength, rather than on the exact reproduction of absolute strength values for a particular sand–structure interface. The corresponding verification scheme is shown in Fig 3.

#### 3.1.1. Parameter calibration.

The proposed model contains nine parameters: the critical-state-line parameters $e_{\Gamma }$, λ, and ξ; the constant-volume friction angle $\varphi_{cv}$; the dilatancy coefficient A; the normal constraint stiffness $K_{n}$; the tangential elastic stiffness $K_{s}$; the shear-band thickness t; and the normal tensile strength $\sigma_{t}$. These parameters have distinct physical meanings and can be determined through critical-state relationships, interface shear responses, CNS boundary conditions, and interface opening characteristics. A summary of their definitions and calibration methods is provided below. Based on the test scheme and interface response characteristics reported by Porcino et al. [4], the parameter calibration follows the principles described below.

1. The critical-state-line parameters $e_{\Gamma }$, λ, and ξ are used to describe the variation in the critical-state void ratio of dense sand under different normal stress levels. Their physical meanings originate from the framework of critical-state soil mechanics. If drained triaxial test results or critical-state-line data are available for the tested sand, these parameters can be directly adopted. Otherwise, they may be estimated from the stabilized volumetric response and residual strength level observed in the later stage of interface shearing.
2. The constant-volume friction angle $\varphi_{cv}$ is determined from the stable frictional response at large interface displacement. Since the proposed model attributes the additional pre-peak strengthening mainly to the contribution of dilatancy, $\varphi_{cv}$ should represent the basic friction level of the interface when the material approaches the critical state and no longer exhibits significant volumetric deformation.
3. The normal constraint stiffness $K_{n}$ is taken from the CNS boundary condition used in the tests of Porcino et al. [4]. This parameter represents the restriction imposed on interface normal expansion and controls the conversion of dilatancy-induced normal deformation into a normal stress increment.
4. The tangential elastic stiffness $K_{s}$ is determined from the slope of the initial approximately linear segment of the shear stress-tangential displacement curve. This parameter controls the elastic response before yielding and is also used to calculate the plastic tangential displacement increment after yielding.
5. The dilatancy coefficient A is the key coupling parameter of the proposed model. It controls the conversion amplitude from the state parameter Ψ to the dilatancy rate D. The calibration of A should not rely solely on the peak shear strength, but should be conducted by jointly fitting the shear stress-tangential displacement curve and the normal stress-tangential displacement curve. Specifically, the initial range of A can be estimated from the pre-peak rate of normal stress increase, and then adjusted according to the peak position, the degree of post-peak softening, and the final stable value of normal stress.
6. The shear-band thickness t is treated as an equivalent thickness of the localized deformation zone near the interface. Since the interface tests of Porcino et al. [4] mainly reflect local rearrangement of sand particles near the structural surface, t can be selected with reference to the interface shear-band thickness observed by DeJong et al. [5] using PIV measurements. In general, t may be taken as several times the mean particle diameter $d_{\text{5}\text{0}}$ and adjusted within a reasonable range so that the calculated void-ratio evolution is consistent with the magnitude of the normal plastic displacement.
7. The normal tensile strength $\sigma_{t}$ is introduced to control possible interface opening and separation. For sand-structure interfaces under compression-shear conditions, this parameter is usually small and may be specified according to interface bonding characteristics, experimental observations, or numerical stability requirements. Once the updated normal stress enters the tensile region and exceeds this limit, the interface is regarded as open, and both the normal stress and shear stress are set to zero.

The parameter calibration procedure is conducted sequentially. First, the critical-state-line parameters ($e_{\Gamma }$, λ, and ξ) are determined from critical-state information or the stabilized response at large displacement. Then, the constant-volume friction angle ($\varphi_{cv}$) is calibrated based on the residual shear strength, while the stiffness parameters ($K_{s}$ and $K_{n}$) are obtained from the initial linear portions of the shear stress–displacement and normal stress–displacement curves, respectively. The dilatancy coefficient (A) is calibrated by jointly considering the pre-peak shear response, normal stress evolution, peak strength, and post-peak softening behavior. Finally, the shear-band thickness (t) and normal tensile strength ($\sigma_{t}$) are determined according to the deformation characteristics and interface opening condition. When normal-stress measurements are unavailable, A can be primarily calibrated from the shear stress–displacement response, although the accuracy of dilatancy-related parameters may be reduced.

Therefore, the parameter-calibration strategy combines direct assignment of test boundary conditions, physical constraints from critical-state parameters, and joint calibration based on shear and normal stress responses. This approach preserves the physical meaning of the model parameters and reduces dependence on purely empirical fitting.

#### 3.1.2. Verification results and analysis.

**Fig 4:**
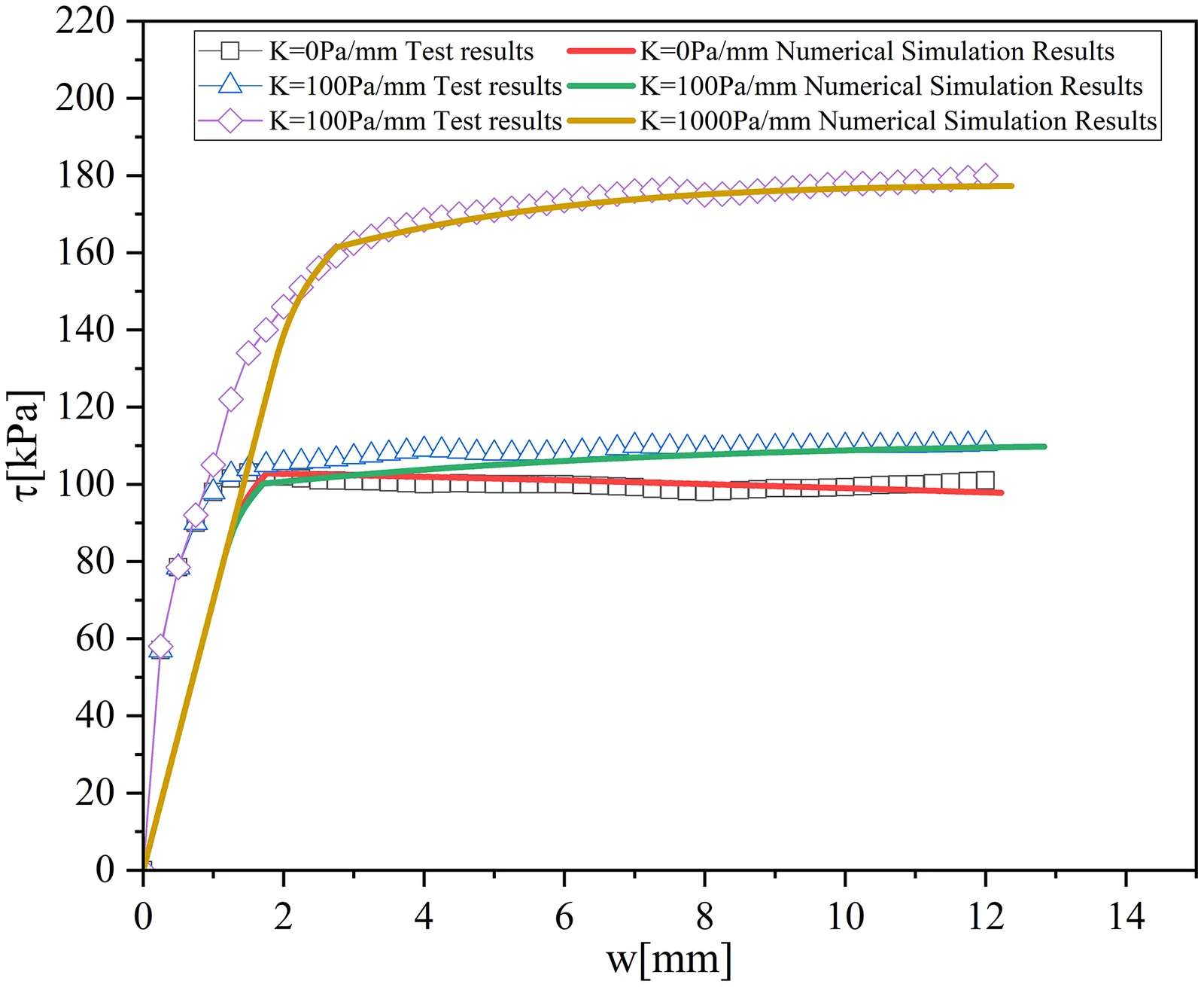
Interface shear stress–tangential displacement relationship: comparison between model prediction and test results.

Fig 4 compares the model predictions with the CNS direct-shear test results reported by Porcino et al. [4] in terms of the interface shear stress–tangential displacement relationship. The comparisons indicate that the proposed model can qualitatively reproduce the main response characteristics of interface shearing, including the evolution of shear stress, normal stress increase, and post-peak softening behavior. At the initial stage of shearing, the shear stress increases rapidly with tangential displacement, reflecting the combined effects of interface friction mobilization and dilatancy-induced normal-stress increase. With further shearing, the shear stress gradually reaches a peak and subsequently decreases toward a residual level, indicating that the model is capable of capturing the complete evolution process from pre-peak strengthening to post-peak softening. Overall, the predicted curve agrees well with the test results in terms of peak position, post-peak softening trend, and overall curve shape, which supports the rationality of using the state parameter to control dilatancy evolution and the CNS constraint to describe normal-stress development.

### 3.2. Parametric sensitivity analysis under CNS interface conditions

To further clarify the influence of key parameters in the proposed state-dependent dilatancy-coupled interface model on the shearing response of dense sand-structure interfaces, a parametric sensitivity analysis is conducted based on the CNS direct-shear verification condition presented in Section 3.1. The case with a CNS normal constraint stiffness of K=1000 kPa/mm is selected as the baseline case. A single-factor variation method is adopted, in which one parameter is varied at a time while the remaining parameters are kept unchanged.

The analysis focuses on the effects of the critical-state-line parameters $e_{\Gamma }$, λ, and ξ, the dilatancy coefficient A, the shear-band thickness t, and the initial void ratio $e_{\text{0}}$ on the interface shear stress-tangential displacement response. In this section, the CNS boundary stiffness is kept constant to eliminate the influence of changes in external normal constraint. This allows the effects of the internal state parameters and dilatancy-control parameters on peak strength, post-peak softening, and curve shape to be examined more clearly.

#### 3.2.1. Influence of critical-state-line parameters.

**Fig 5:**
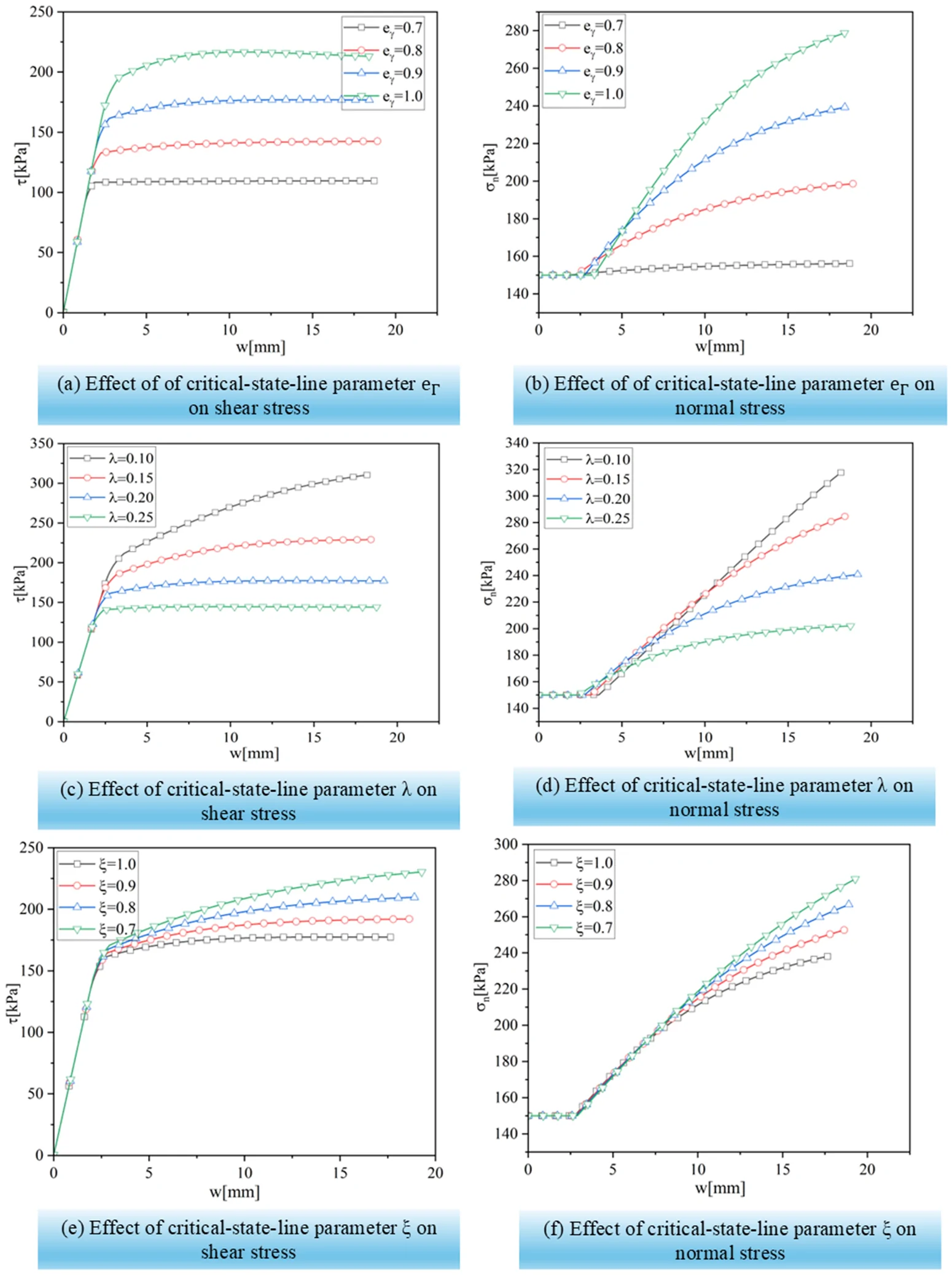
Effects of critical-state line parameters on the shear stress and normal stress responses of the dense-sand pile–soil interface under CNS boundary conditions.

The critical-state-line parameters $e_{\Gamma }$, λ, and ξ describe the variation in the critical-state void ratio of sand under different normal stress levels and provide the basis for calculating the state parameter. In the proposed model, the state parameter is defined as the difference between the current void ratio e and the critical-state void ratio $e_{c}$ under the same normal stress, namely $\Psi =e-e_{c}.$ Therefore, when the initial void ratio and normal constraint stiffness remain unchanged, variations in the critical-state-line parameters directly change the state parameter, thereby affecting the dilatancy potential, the magnitude of normal-stress increase, and the interface shear strength. Fig 5 shows the variations in interface shear stress and normal stress with tangential displacement under different values of the critical-state-line parameters.

As $e_{\Gamma }$ increases, the critical-state void ratio increases, and the interface material becomes relatively denser with respect to the critical state. As a result, the dilatancy tendency during shearing is enhanced. This leads to a marked increase in the shear stress level and a larger normal-stress increment, indicating that $e_{\Gamma }$ mainly affects the interface response by modifying the initial dilatancy potential.

As λ increases, both the interface shear stress and normal stress generally decrease. This indicates that, within the parameter range considered in this study, a larger λ reduces the dense-state tendency of the interface material and weakens its dilatancy capacity. Under CNS conditions, the normal-stress increment induced by constrained dilatancy is consequently reduced, which further lowers the mobilized interface shear strength.

A similar decreasing trend is observed when ξ increases. This suggests that ξ affects the sensitivity of the critical-state void ratio to changes in normal stress, thereby altering the state parameter and the corresponding dilatancy response. Overall, the critical-state-line parameters do not directly change the basic frictional property of the interface. Instead, they indirectly influence dilatancy-induced strengthening and normal-stress development by regulating the state parameter.

#### 3.2.2. Influence of the dilatancy coefficient.

**Fig 6:**
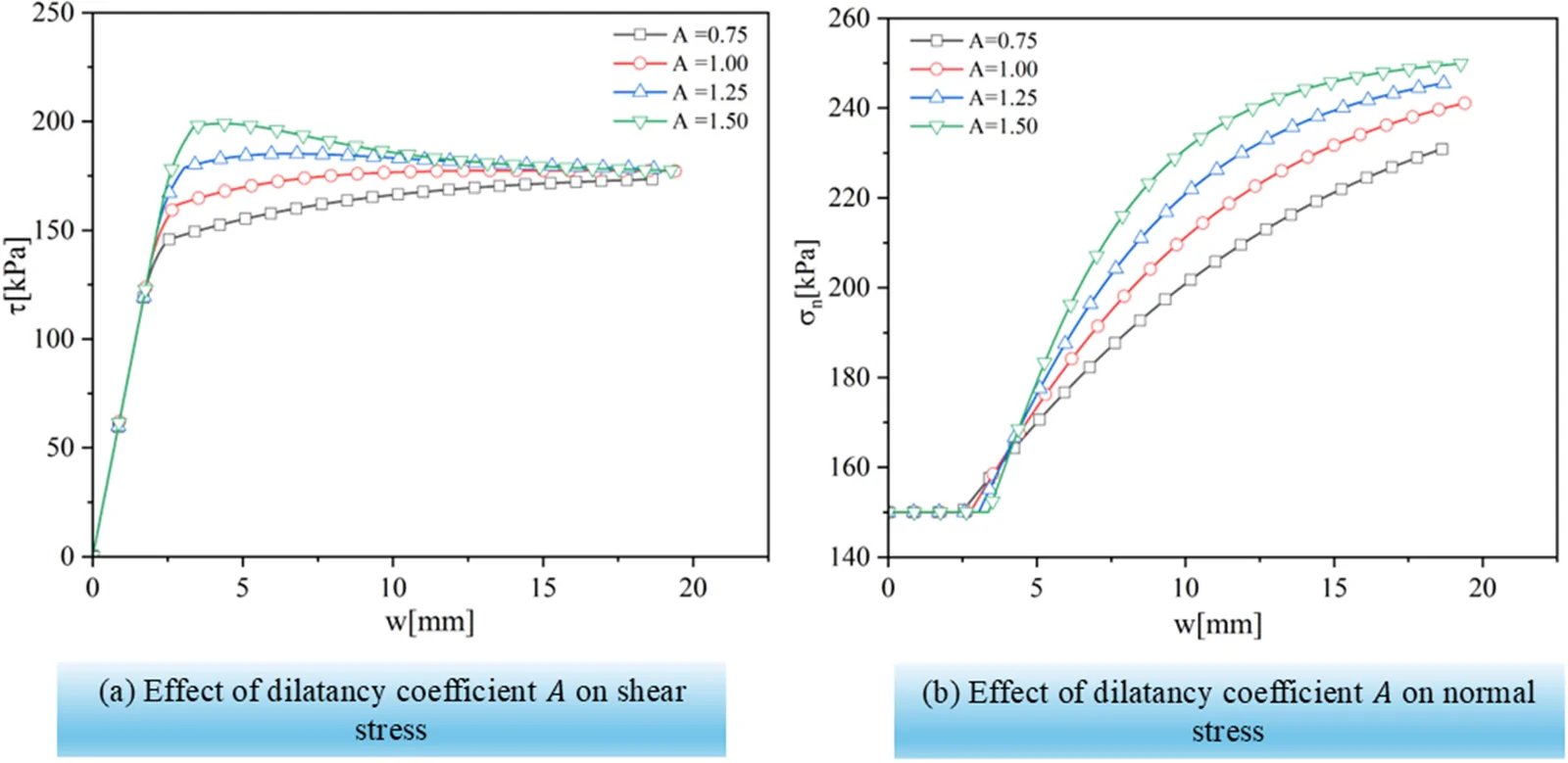
Effects of dilatancy coefficient on the shear stress and normal stress responses of the dense-sand pile–soil interface under CNS boundary conditions.

The dilatancy coefficient A controls the conversion amplitude from the state parameter Ψ to the dilatancy rate D, and is therefore a key parameter governing dilatancy-induced strengthening of the interface. As shown in Fig 6, with increasing A, the peak interface shear stress increases significantly, and the magnitude of normal-stress increase also becomes more pronounced. This is because a larger dilatancy coefficient allows the same plastic tangential displacement to produce a larger normal plastic displacement. Under the CNS constraint, this normal expansion tendency is converted into an additional normal stress increment, which further enhances the mobilized shear strength of the interface.

When A is relatively small, the interface response is closer to ordinary frictional sliding, and the dilatancy-induced strengthening effect is limited. In contrast, when A is larger, the peak characteristics of the shear stress-displacement curve become more evident, and more pronounced post-peak softening may occur due to the subsequent attenuation of dilatancy. Therefore, the dilatancy coefficient affects not only the peak shear strength, but also the rate of normal-stress development and the post-peak strength evolution of dense sand-structure interfaces under CNS conditions.

#### 3.2.3. Influence of shear-band thickness.

**Fig 7:**
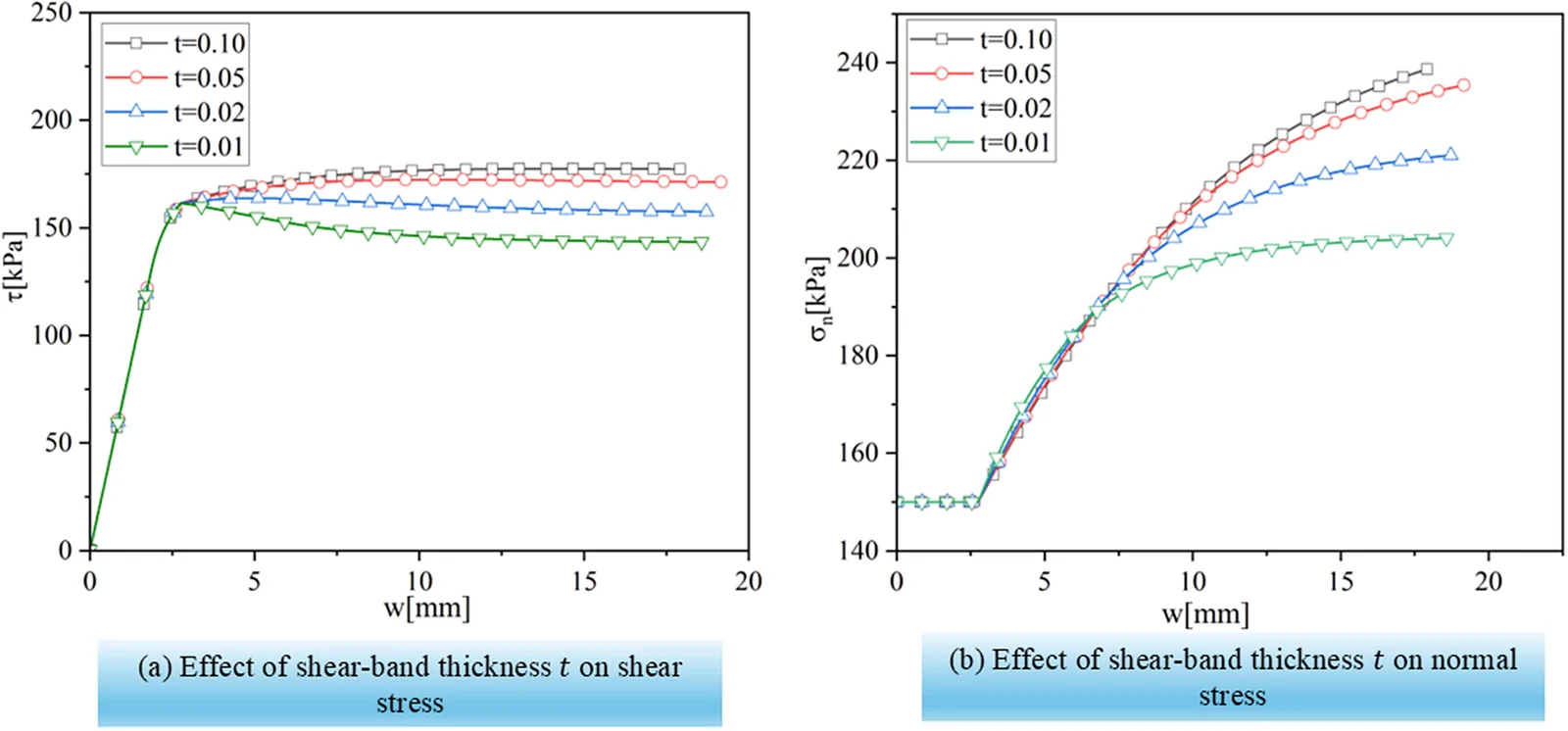
Influence of shear-band thickness on the shear stress and normal stress responses of the dense-sand pile–soil interface under CNS boundary conditions.

The shear-band thickness 𝑡 represents the effective range of localized shear deformation near the interface and controls the rate of void-ratio evolution induced by normal plastic deformation. As shown in Fig 7, the shear-band thickness has a noticeable influence on both the shape of the shear stress–displacement curve and the development of normal stress. When 𝑡 is relatively small, the same normal plastic displacement produces a larger change in the void ratio within the interface shear band. As a result, the state parameter evolves more rapidly toward the critical state, and the dilatancy capacity attenuates earlier. This causes the shear stress to soften more readily after the peak, while the magnitude of normal-stress increase remains relatively limited. In contrast, when 𝑡 increases, the void-ratio evolution becomes slower, allowing dilatancy to be maintained over a larger range of tangential displacement. Consequently, both the shear stress and normal stress remain at higher levels, and the corresponding curves become smoother. Therefore, the shear-band thickness mainly affects the post-peak softening behavior by controlling the rate of state evolution within the interface shear band.

#### 3.2.4. Influence of initial void ratio.

**Fig 8:**
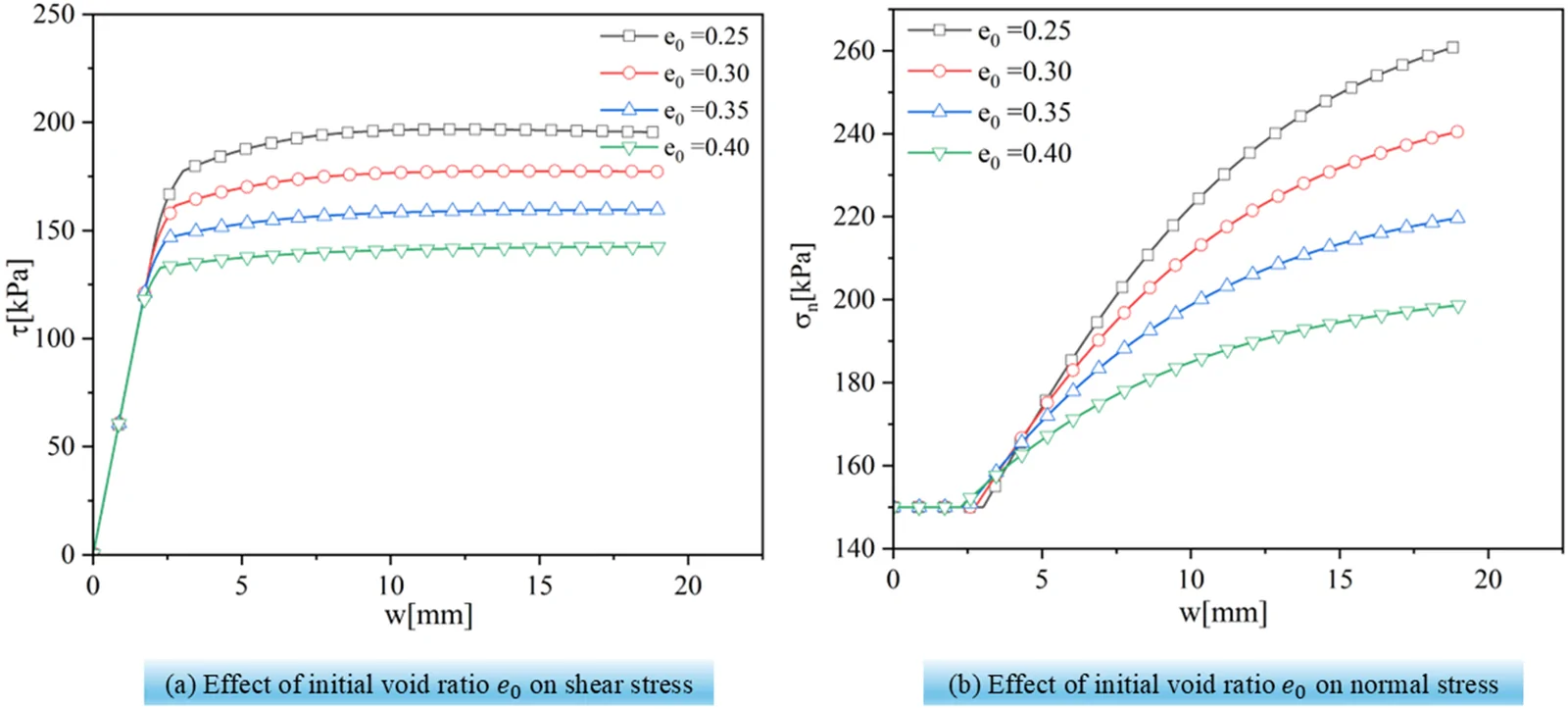
Influence of initial void ratio on the shear stress and normal stress responses of the dense-sand pile–soil interface under CNS boundary conditions.

The initial void ratio $e_{\text{0}}$ reflects the initial density state of the sand within the interface shear band and is an important state variable controlling the dilatancy potential of dense sand-structure interfaces. As shown in Fig 8, a smaller initial void ratio leads to a higher interface shear stress and a more pronounced increase in normal stress. This is because a lower $e_{\text{0}}$ corresponds to a denser interface state, in which particle interlocking and rearrangement during shearing are more significant, resulting in a stronger tendency for dilatancy.

Under the CNS constraint, the normal expansion induced by dilatancy is converted into an additional normal-stress increment, thereby enhancing the mobilized shear strength of the interface. As $e_{\text{0}}$ increases, the interface material becomes closer to the critical state, and its dilatancy potential gradually weakens. Consequently, both the normal-stress increase and the dilatancy-induced strengthening effect decrease, leading to less pronounced peak strength and post-peak softening characteristics in the shear stress-displacement curve.

#### 3.2.5. Comprehensive analysis of parameter sensitivity.

The above results indicate that, under CNS conditions, different parameters affect the shearing response of dense sand–structure interfaces through different mechanisms. The critical-state-line parameters and the initial void ratio mainly control the initial dilatancy potential by influencing the state parameter. The dilatancy coefficient directly determines the magnitude of the dilatancy rate and is the key parameter governing the peak shear strength and the magnitude of normal-stress increase. The shear-band thickness primarily affects the rate of void-ratio evolution within the interface shear band, thereby influencing post-peak softening and the smoothness of the stress–displacement curves.

Overall, the dilatancy coefficient and the initial void ratio have the most direct influence on the interface strength level. The shear-band thickness has a more pronounced effect on post-peak softening and state evolution, whereas the critical-state-line parameters indirectly regulate the dilatancy-induced strengthening effect by changing the critical-state void ratio and the corresponding state parameter. These results demonstrate that the proposed model can reasonably capture the coupled evolution mechanism of dilatancy, normal-stress increase, strength mobilization, and dilatancy attenuation for dense sand–structure interfaces under CNS conditions.

## 4. Conclusions

This paper develops a state-dependent dilatancy-coupled constitutive model for dense sand–structure interfaces under CNS conditions, aiming to describe the coupled response of constrained dilatancy, normal-stress increase, peak-strength mobilization, and post-peak softening during interface shearing. The proposed model is verified against CNS direct-shear test results, and the effects of key parameters are further investigated through parametric sensitivity analysis. The main conclusions are as follows:

(1) Based on the state-parameter concept in critical-state soil mechanics, a density-state-dependent dilatancy evolution relationship is established for dense sand–structure interfaces. The model links particle rearrangement, dilatancy attenuation, and shear-strength evolution, and can describe the continuous process of initial dilatancy, constrained normal-stress increase, peak-strength formation, dilatancy attenuation, and post-peak softening.
(2) By introducing the CNS boundary condition, the model couples shear-induced dilatancy with normal-stress evolution. The normal plastic deformation generated during interface shearing is converted into an increment of normal stress under the imposed normal constraint, thereby enhancing the mobilized shear strength. As the state parameter gradually evolves toward the critical state, the dilatancy capacity attenuates, and the interface strength gradually transitions from the peak value to a stable residual level.
(3) Verification based on CNS direct-shear test results shows that the proposed model can reasonably reproduce the typical shear response of dense sand–structure interfaces, including the rapid increase in shear stress at the initial stage, peak-strength mobilization, and post-peak softening. This confirms the rationality of using the state parameter to control dilatancy evolution and the CNS constraint to describe normal-stress development.
(4) The parametric sensitivity analysis indicates that the critical-state-line parameters and the initial void ratio mainly affect the initial dilatancy potential by changing the state parameter. The dilatancy coefficient directly controls the magnitude of the dilatancy rate and is the key parameter influencing the peak shear strength and normal-stress increase. The shear-band thickness mainly affects the rate of void-ratio evolution within the interface shear band and thereby regulates post-peak softening and curve smoothness.
(5) Overall, the proposed model uses a limited number of parameters with clear physical meanings and can effectively describe the coupled evolution of density state, dilatancy, normal stress, and shear strength of dense sand–structure interfaces under CNS conditions. The model provides a concise constitutive framework for analyzing the mechanical response of dense sand–structure interfaces and for further interface-level numerical simulations.

## Supporting information

S1 File Simulation data. (OPJU)

S2 File Validated. (OPJU)
